# The impact of sports participation on mental health and social outcomes in adults: a systematic review and the ‘Mental Health through Sport’ conceptual model

**DOI:** 10.1186/s13643-023-02264-8

**Published:** 2023-06-21

**Authors:** Narelle Eather, Levi Wade, Aurélie Pankowiak, Rochelle Eime

**Affiliations:** 1 Centre for Active Living and Learning, University of Newcastle, University Drive, Callaghan, NSW 2308 Australia; 2 College of Human and Social Futures, School of Education, University of Newcastle, University Drive, Callaghan, NSW 2308 Australia; 3 College of Health, Medicine, and Wellbeing, School of Medicine and Public Health, University of Newcastle, University Drive, Callaghan, NSW 2308 Australia; 4 Institute for Health and Sport, Victoria University, Ballarat Road, Footscray, VIC 3011 Australia; 5 School of Science, Psychology and Sport, Federation University Australia, University Drive, Mount Helen, VIC 3350 Australia

**Keywords:** Adults, Review, Experimental, Observational, Psychological health, Mental health, Social health, Sport, Model

## Abstract

**Background:**

Sport is a subset of physical activity that can be particularly beneficial for short-and-long-term physical and mental health, and social outcomes in adults. This study presents the results of an updated systematic review of the mental health and social outcomes of community and elite-level sport participation for adults. The findings have informed the development of the ‘Mental Health through Sport’ conceptual model for adults.

**Methods:**

Nine electronic databases were searched, with studies published between 2012 and March 2020 screened for inclusion. Eligible qualitative and quantitative studies reported on the relationship between sport participation and mental health and/or social outcomes in adult populations. Risk of bias (ROB) was determined using the Quality Assessment Tool (quantitative studies) or Critical Appraisal Skills Programme (qualitative studies).

**Results:**

The search strategy located 8528 articles, of which, 29 involving adults 18–84 years were included for analysis. Data was extracted for demographics, methodology, and study outcomes, and results presented according to study design. The evidence indicates that participation in sport (community and elite) is related to better mental health, including improved psychological well-being (for example, higher self-esteem and life satisfaction) and lower psychological ill-being (for example, reduced levels of depression, anxiety, and stress), and improved social outcomes (for example, improved self-control, pro-social behavior, interpersonal communication, and fostering a sense of belonging). Overall, adults participating in team sport had more favorable health outcomes than those participating in individual sport, and those participating in sports more often generally report the greatest benefits; however, some evidence suggests that adults in elite sport may experience higher levels of psychological distress. Low ROB was observed for qualitative studies, but quantitative studies demonstrated inconsistencies in methodological quality.

**Conclusions:**

The findings of this review confirm that participation in sport of any form (team or individual) is beneficial for improving mental health and social outcomes amongst adults. Team sports, however, may provide more potent and additional benefits for mental and social outcomes across adulthood. This review also provides preliminary evidence for the Mental Health through Sport model, though further experimental and longitudinal evidence is needed to establish the mechanisms responsible for sports effect on mental health and moderators of intervention effects. Additional qualitative work is also required to gain a better understanding of the relationship between specific elements of the sporting environment and mental health and social outcomes in adult participants.

**Supplementary Information:**

The online version contains supplementary material available at 10.1186/s13643-023-02264-8.

## Introduction

The organizational structure of sport and the performance demands characteristic of sport training and competition provide a unique opportunity for participants to engage in health-enhancing physical activity of varied intensity, duration, and mode; and the opportunity to do so with other people as part of a team and/or club. Participation in individual and team sports have shown to be beneficial to physical, social, psychological, and cognitive health outcomes [1–7]. Often, the social and mental health benefits facilitated through participation in sport exceed those achieved through participation in other leisure-time or recreational activities [8–10]. Notably, these benefits are observed across different sports and sub-populations (including youth, adults, older adults, males, and females) [11]. However, the evidence regarding sports participation at the elite level is limited, with available research indicating that elite athletes may be more susceptible to mental health problems, potentially due to the intense mental and physical demands placed on elite athletes [12].

Participation in sport varies across the lifespan, with children representing the largest cohort to engage in organized community sport [13]. Across adolescence and into young adulthood, dropout from organized sport is common, and especially for females [14–16], and adults are shifting from organized sports towards leisure and fitness activities, where individual activities (including swimming, walking, and cycling) are the most popular [13, 17–19]. Despite the general decline in sport participation with age [13], the most recent (pre-COVID) global data highlights that a range of organized team sports (such as, basketball, netball volleyball, and tennis) continue to rank highly amongst adult sport participants, with soccer remaining a popular choice across all regions of the world [13]. It is encouraging many adults continue to participate in sport and physical activities throughout their lives; however, high rates of dropout in youth sport and non-participation amongst adults means that many individuals may be missing the opportunity to reap the potential health benefits associated with participation in sport.

According to the World Health Organization, mental health refers to a state of well-being and effective functioning in which an individual realizes his or her own abilities, is resilient to the stresses of life, and is able to make a positive contribution to his or her community [20]. Mental health covers three main components, including psychological, emotional and social health [21]. Further, psychological health has two distinct indicators, psychological well-being (e.g., self-esteem and quality of life) and psychological ill-being (e.g., pre-clinical psychological states such as psychological difficulties and high levels of stress) [22]. Emotional well-being describes how an individual feels about themselves (including life satisfaction, interest in life, loneliness, and happiness); and social well–being includes an individual’s contribution to, and integration in society [23].

Mental illnesses are common among adults and incidence rates have remained consistently high over the past 25 years (~ 10% of people affected globally) [24]. Recent statistics released by the World Health Organization indicate that depression and anxiety are the most common mental disorders, affecting an estimated 264 million people, ranking as one of the main causes of disability worldwide [25, 26]. Specific elements of social health, including high levels of isolation and loneliness among adults, are now also considered a serious public health concern due to the strong connections with ill-health [27]. Participation in sport has shown to positively impact mental and social health status, with a previous systematic review by Eime et al. (2013) indicated that sports participation was associated with lower levels of perceived stress, and improved vitality, social functioning, mental health, and life satisfaction [1]. Based on their findings, the authors developed a conceptual model (health through sport) depicting the relationship between determinants of adult sports participation and physical, psychological, and social health benefits of participation. In support of Eime’s review findings, Malm and colleagues (2019) recently described how sport aids in preventing or alleviating mental illness, including depressive symptoms and anxiety or stress-related disease [7]. Andersen (2019) also highlighted that team sports participation is associated with decreased rates of depression and anxiety [11]. In general, these reviews report stronger effects for sports participation compared to other types of physical activity, and a dose–response relationship between sports participation and mental health outcomes (i.e., higher volume and/or intensity of participation being associated with greater health benefits) when adults participate in sports they enjoy and choose [1, 7]. Sport is typically more social than other forms of physical activity, including enhanced social connectedness, social support, peer bonding, and club support, which may provide some explanation as to why sport appears to be especially beneficial to mental and social health [28].

Thoits (2011) proposed several potential mechanisms through which social relationships and social support improve physical and psychological well-being [29]; however, these mechanisms have yet to be explored in the context of sports participation at any level in adults. The identification of the mechanisms responsible for such effects may direct future research in this area and help inform future policy and practice in the delivery of sport to enhance mental health and social outcomes amongst adult participants. Therefore, the primary objective of this review was to examine and synthesize all research findings regarding the relationship between sports participation, mental health and social outcomes at the community and elite level in adults. Based on the review findings, the secondary objective was to develop the ‘Mental Health through Sport’ conceptual model.

## Methods

This review has been registered in the PROSPERO systematic review database and assigned the identifier: CRD42020185412. The conduct and reporting of this systematic review also follows the Preferred Reporting for Systematic Reviews and Meta-Analyses (PRISMA) guidelines [30] (PRISMA flow diagram and PRISMA Checklist available in supplementary files). This review is an update of a previous review of the same topic [31], published in 2012.

### Identification of studies

Nine electronic databases (CINAHL, Cochrane Library, Google Scholar, Informit, Medline, PsychINFO, Psychology and Behavioural Sciences Collection, Scopus, and SPORTDiscus) were systematically searched for relevant records published from 2012 to March 10, 2020. The following key terms were developed by all members of the research team (and guided by previous reviews) and entered into these databases by author LW: sport* AND health AND value OR benefit* OR effect* OR outcome* OR impact* AND psych* OR depress* OR stress OR anxiety OR happiness OR mood OR ‘quality of life’ OR ‘social health’ OR ‘social relation*’ OR well* OR ‘social connect*’ OR ‘social functioning’ OR ‘life satisfac*’ OR ‘mental health’ OR social OR sociolog* OR affect* OR enjoy* OR fun. Where possible, Medical Subject Headings (MeSH) were also used.

### Criteria for inclusion/exclusion

The titles of studies identified using this method were screened by LW. Abstract and full text of the articles were reviewed independently by LW and NE. To be included in the current review, each study needed to meet each of the following criteria: (1) published in English from 2012 to 2020; (2) full-text available online; (3) original research or report published in a peer-reviewed journal; (4) provides data on the psychological or social effects of participation in sport (with sport defined as a subset of exercise that can be undertaken individually or as a part of a team, where participants adhere to a common set of rules or expectations, and a defined goal exists); (5) the population of interest were adults (18 years and older) and were apparently healthy. All papers retrieved in the initial search were assessed for eligibility by title and abstract. In cases where a study could not be included or excluded via their title and abstract, the full text of the article was reviewed independently by two of the authors.

### Data extraction

For the included studies, the following data was extracted independently by LW and checked by NE using a customized Google Docs spreadsheet: author name, year of publication, country, study design, aim, type of sport (e.g., tennis, hockey, team, individual), study conditions/comparisons, sample size, where participants were recruited from, mean age of participants, measure of sports participation, measure of physical activity, psychological and/or social outcome/s, measure of psychological and/or social outcome/s, statistical method of analysis, changes in physical activity or sports participation, and the psychological and/or social results.

### Risk of bias (ROB) assessment

A risk of bias was performed by LW and AP independently using the ‘Quality Assessment Tool for Observational Cohort and Cross-Sectional Studies’ OR the ‘Quality Assessment of Controlled Intervention Studies’ for the included quantitative studies, and the ‘Critical Appraisal Skills Programme (CASP) Checklist for the included qualitative studies [32, 33]. Any discrepancies in the ROB assessments were discussed between the two reviewers, and a consensus reached.

## Results

**Fig. 1:**
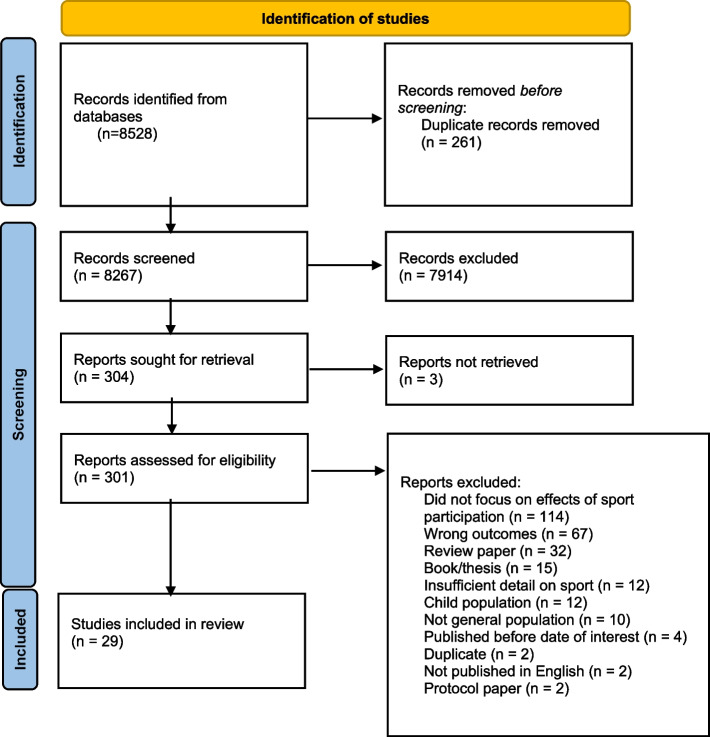
Flow of studies through the review process

The search yielded 8528 studies, with a total of 29 studies included in the systematic review (Fig. 1). Tables 1 and 2 provide a summary of the included studies. The research included adults from 18 to 84 years old, with most of the evidence coming from studies targeting young adults (18–25 years). Study samples ranged from 14 to 131, 962, with the most reported psychological outcomes being self-rated mental health (*n* = 5) and depression (*n* = 5). Most studies did not investigate or report the link between a particular sport and a specific mental health or social outcome; instead, the authors’ focused on comparing the impact of sport to physical activity, and/or individual sports compared to team sports. The results of this review are summarized in the following section, with findings presented by study design (cross-sectional, experimental, and longitudinal).

**Table 1:** Studies on the relationship between sport and social outcomes

Social outcomes						
1. Author 2. Study design	Aim	Sport 1. Mode 2. Structure	Conditions/comparison	1. Sample size (*n*) 2. Sex ratio 3. Mean age (SD)/age range	Psychological outcome/s and measure	Key findings
1. Brinkley, 2017 [34] 2. Quantitative Quasi-experimental Non-randomized	To examine the impact of participating in 12-week workplace team sport on VO2 max, and of individual, social group, and organizational health	1. Rounders, netball, basketball, soccer, cricket, and handball 2. Team	*Intervention:* 12 × weekly lunchtime moderate-intensity team sport sessions in an indoor sports hall (10 min warm-up, 40 min of sport) *Control:* normal work conditions	1. 48 2. 28 males: 20 females aged 24 to 64 *Intervention* *n* = 28; mean = 39.59 (9.11) *Control* 1. *n* = 20; mean = 40.75 (11.92)	Social cohesion Sub-scales from the Copenhagen Psychosocial Questionnaire-II Interpersonal communication (relationships with colleagues) Climate uniformity Interpersonal relationships (with superiors) Copenhagen Psychosocial Questionnaire-II-II	There were significant improvements in interpersonal communication favoring the intervention group, though there were no other significant effects
1. Dore, 2018 [35] 2. Quantitative-longitudinal (6 months) cross-sectional	This study examined the associations between the context of physical activity (PA) is undertaken (team sports, informal group, individual PA), and positive mental health, anxiety symptoms and depressive symptoms. It also investigated whether social connectedness or PA volume mediate these associations	1. Team sports 2. Team	Team sports vs. informal group PA vs. individual PA	1. 1446 2. 602 males: 844 females 3. mean age = 18.4 (2.4)	Social connectedness in PA Relatedness to Others in Physical Activity Scale (ROPAS)	Relative to those who engaged in individual PA, being active in informal group PA or sports team was associated with higher levels of social connectedness and higher moderate-vigorous PA volume. In mediation analysis, the hypothesis that PA context influences mental health indirectly through social connectedness was not confirmed
1. Marlier, 2015 [36] 2. Quantitative cross-sectional	The study aimed to uncover how sport participation, physical activity, social capital, and mental health are interrelated	1. Sport participation-organized and non-organized 2. Team and individual	Examined how sport participation, total physical activity, social capital, and mental health are interrelated	1. 414 2. – 3. age range 18 to 56 years	Social capital (individual, and community) evaluated using a scale based on the ‘social capital community benchmark survey’ Thematic analysis (interview)	No direct associations were found between sport participation and community social capital (or individual social capital Individual social capital (but not community social capital) had a direct association with mental health. A significant indirect association of community social capital with mental health was discovered through individual social capital (i.e., individual social capital partially mediates the relation between community social capital and mental health)
1. McGraw, 2018 [37] 2. Qualitative	To study how NFL players and their family members characterize the impact of an NFL career on the mental and emotional health of NFL players	1. NFL (American football) 2. Team	Includes both current and former NFL players, and family members of these players	1. 25 (23 former and 2 current) players, 27 family members (88.9% wife; 3.7% parent; and 7.4% were 'other' (child, sibling) 2. All male 3.Age not mentioned	**Social isolation** Thematic analysis (interview)	The players and their families noted concerns related to making and keeping social connections with people outside of the NFL; isolation due to physical size, celebrity status, team roster turnover; racial, socioeconomic, and cultural differences among players; loss of their social connection with the team and teammates through practices and games. Of note is the paradoxical situation that although football is a team sport, individual players can feel very much alone due to the nature and structure of the sport
1. Mickelsson, 2020 [38] 2. Quantitative—longitudinal (5 months)	The current study sought to explore how mixed martial-arts (MMA) and Brazilian jiu-jitsu (BJJ) affect deviant and socially desirable traits among adolescents and young adults who were new to the sports and how individuals predisposed to certain traits may be more inclined to train in one sport than another	Mixed Martial Arts (MMA) and Brazilian Jiu-Jitsu (BJJ)	Jiu-jitsu vs mixed-martial arts	1. 113 2. 105 males: 8 females 3. No age data	Aggression Buss-Perry Aggression Questionnaire (BPAQ) Pro-social behavior Pro-socialness Scale for Adults (PSA) Self-control Self-Control Scale (SCS) Criminal behavior (frequency) Total Delinquent Acts Measure (TDAM)	MMA practitioners slightly increased their levels of aggression, BJJ practitioners reduced theirs. Both MMA and BJJ increased self-control and pro-social behavior; and individuals who sought out to practice MMA were more predisposed to having higher levels of aggression. BJJ practitioners developed more pro-social behaviour and reduced aggression compared to MMA practitioners; but there was no significant interaction between self-control and sport as self-control improved among all practitioners. No interaction between sport and crime frequency was found, with both groups reducing criminal behavior moderately (but low baseline scores)
1. Purcell, 2020 [39] 2. Quantitative cross-sectional	The purpose of this study was to assess the prevalence and correlates of mental health symptoms in a representative, national sample of elite athletes and to compare rates against published community norms	1. Elite athletes (no singular sport) 2. Not specified	Community rates of psychological variables; 'published community norms'	1. 749 2. 344 males 3. mean age = 24.6 (sd = 6.9)	Social dysfunction Subscale of the GHQ 28	There was no relationship between social dysfunction and sport participation
1. Thorpe, 2014 [40] 2. Qualitative	The aim of the study was to understand the impact of an Aboriginal community sporting team and its environment on the social, emotional, and physical wellbeing of young Aboriginal men	1. Australian Rules Football 2. Team	–	1. 14 2. All men 3. Age not provided	Mental well-being Social, emotional, and physical wellbeing impact (4 semi structured interview; 3 focus groups)	Some respondents felt social and community aspects of participating were just as important to players as individual health benefits gained from participation. Participants felt a sense of ownership and belonging when playing with an Aboriginal sports team. The club was also a place they felt safe and comfortable

**Table 2:** Studies on the relationship between sport and mental health

Psychological outcomes						
1. Author 2. Study design (qual/quant)	Aim	Sport 1. Mode 2. Structure	Conditions/comparison	1. Sample Size 2. Male/female ratio 3. Mean age (SD)	Psychological outcome/s and measure	Key findings
1. Appelqvist-Schmidlechner, 2018 [41] 2. Quantitative-cross-sectional (retroactive)	To examine whether retrospectively assessed sports participation (SP) and competitive sports (CS) at the age of 12 years is associated with mental health and health behavior in young adulthood among males	1. Structured sport participation at age 12 2. Not specified	No participation in sport at age 12	1. 680 2. 100% male 3. 26 years (4)	Mental well-being Warwick-Edinburgh Mental Well-Being Scale (short version) Mental distress The Short Form Health Survey (SF-36) scale ('5 items')	Sports participation (SP) at 12 years is associated with better mental health in young adulthood-mental wellbeing and mental distress. This was also the case when controlling the impact of age, education, and present leisure-time physical activity. Higher level of intensity of SP and the level of competitive sport in childhood were associated with lower level of mental distress in adulthood
1. Ashdown-Franks, 2017 [42] 2. Quantitative—longitudinal	To examine the longitudinal associations between sport participation during high school and symptoms of panic disorder, GAD, social phobia, and agoraphobia in young adulthood (assessed 3 years after high school)	1. 18 different sports 2. Individual and team	Years of participation in 18 different sports (yes/no)	1. 781 2. 44.8% male 3. 20.34 (0.71)	Anxiety (Panic disorder, generalized anxiety disorder, social phobia, agoraphobia) 2003 Canadian Community Health Survey	The number of years of sport participation in high school was protective of symptoms of panic and agoraphobia in young adulthood, but not protective of symptoms of social phobia or GAD. Participation in either type of sport (individual or team) was protective of panic disorder symptoms, while only team sport was protective of agoraphobia symptoms, and only individual sport was protective of social phobia symptoms
1. Brinkley, 2017 [34] 2. Quantitative-quasi-experimental non-randomized	To investigate the short-term effect of regular sports team participation on individual employee and organizational health, including VO2 max, and individual, social group and organizational health	1. Rounders netball, basketball, soccer, cricket, and handball 2. Team	*Experimental:* 12 × weekly lunchtime moderate-intensity team sport sessions in an indoor sports hall (10 min warm-up, 40 min of sport) ***Control:*** normal work conditions	1. 48 2. 28 males: 20 females 3. Aged 24 to 64	Vitality Subjective vitality scale Quality of life Satisfaction with Life Scale Psychological stress Perceived Stress Scale	Results indicate workplace team sport can improve interpersonal communication within teams, but no significant findings for subjective vitality, quality of life, stress, communication, or relationships (with colleagues or superiors)
1. Brunet, 2013 [43] 2. Longitudinal (prospective)–10 years + cross-sectional	The objectives of this study were to assess (1) the longitudinal associations of past moderate-to-vigorous physical activity (MVPA) and involvement in team sports during secondary school with depressive symptoms in early adulthood, and (2) the cross-sectional associations of current MVPA and involvement in team sports with depressive symptoms during young adulthood	1. Not specified 2. Team	-	1. 860 2.(53.2% female) 3. Mean age at baseline = 12.7, SD = 0.5; mean at survey 21 (end of study)-20.39 (sd = 0.39)	Depressive symptoms The Major Depression Inventory (MDI)	Longitudinal results: There were no statistically significant associations between the MVPA slope in secondary school and depressive symptoms in young adulthood. In contrast, involvement in team sports was significantly and negatively related to depressive symptoms in the univariate model (*P* < .05) Cross-sectional results: significant negative association between current physical activity (i.e., both MVPA and involvement in team sports) and depressive symptoms during young adulthood was observed in the unadjusted linear regression models, but the association between involvement in team sports and depressive symptoms was no longer significant in the adjusted model
1. Chinkov, 2015 [44] 2. Qualitative—interviews (semi-structured)	The purpose of this study was to explore the transfer of life skills among adults who participated in Brazilian jiu-jitsu	1. Brazilian jiu-jitsu 2. Individual	–	1. 14 2. (10/4) 3. Mean age = 34.6 (range 19 to 54)	Self-confidence Individual semi-structured interviews	Twelve of the participants reported that their involvement in Brazilian jiu-jitsu improved their self-confidence outside of the gym. Participants reported improved self-confidence in interactions with others, in being more assertive, and in their ability to defend themselves
1. Ciaccioni, 2019 [45] 2. Quantitative-experimental (non-randomized)	This study aimed to investigate the effects of a 4-month judo training (1 h session, biweekly) on physical and mental health of older adults	1. Judo 2. Individual	*Experimental:* 2 × 60 min sessions weekly for 4 months *Control:* did not receive any training	1. 30 2. (17/13) 3. Mean age = 69.7 (sd = 4.2 years)	Mental Health (includes vitality, social functioning, role-emotional, and mental health) sf12 (v2) Body dissatisfaction Body Image Dimensional Assessment	No significant change in mental health Contrary to what was expected, the 4-month judo program did not affect the body image, or mental health of novice practitioners
1. Dore, 2016 [46] 2. Quantitative-cross-sectional	This study examined the cross-sectional associations among PA volume and context, mental health, and symptoms of anxiety and depression in post-secondary students	1. Team sports 2. Team	Team sports vs. informal group PA vs. individual PA	1. 1446 2. (602/844) 3. Mean age = 18.4 (sd = 2.4)	Mental health The MHC-SF French-Canadian version (Emotional and social wellbeing) Anxiety and depressive symptoms Hospital Anxiety and Depression Scale (HADS)	The findings indicate that total physical activity volume, and moderate-to-vigorous physical activity are positively associated with mental health. Only moderate-to-vigorous physical activity (not total physical activity) was inversely associated with symptoms of anxiety and depression Considering physical activity context, when adjusting for covariates and moderate-to-vigorous physical activity, only participation in team sports (not individual or informal group physical activity) was significantly associated with better mental health
1. Dore, 2018 [35] 2. Quantitative-longitudinal (6 months) cross-sectional	This study examined the associations between the context in which physical activity (PA) is undertaken (team sports, informal group, individual PA), and positive mental health, anxiety symptoms and depressive symptoms. It also investigated whether social connectedness or PA volume mediate these associations	1. Team sports 2. Team	Team sports compared to informal group PA (yoga, running groups etc.) and to individual PA	1. 430 2. 35.5% male 3. Mean age = 18.5 (SD = 2.6)	Mental health Mental Health Continuum-Short Form (MHC-SF) Anxiety and depressive symptoms Hospital Anxiety and Depressive Scale (HADS-A)	Relative to participating in individual physical activity, being involved in either team sports or informal group physical activity was longitudinally associated with better mental health and fewer depressive symptoms Further, team sports were shown to be associated with positive mental health regardless of physical activity volume, suggesting that other qualities of the team sport context contribute to this effect
1. Eime, 2014 [47] 2. Quantitative—cross-sectional	To examine the dose–response relationship between physical activity and health-related quality of life (HRQoL) and life satisfaction. Further, to explore whether these relationships depend on type of physical activity (PA)	1. Membership of a sports club (predominantly tennis and netball) 2. Team	Sports club membership (netball or tennis) vs gymnasium, vs no PA	1. 793 2. All female 3. Mean ages: club (*n* = 499) = 33.9 (SD = 13.7); gym (*n* = 185) = 38.5 (SD = 12.9) = walk (*n* = 109) = 44.5 (SD = 13.2)	Mental health 36-item Short-Form Health Survey (SF-36)—Mental Component Summary (MCS) Life satisfaction Life Satisfaction Score—from the Australian Longitudinal Study on Women’s Health	Compared to people not engaged in sport, sports participants (tennis or netball) reported better mental health and life satisfaction The results showed that there was no dose–response relationship between the level of physical activity (i.e., the amount) and either mental health or life satisfaction, suggesting that factors other than physical activity are responsible for the relationship
1. Gerber, 2014 [48] 2. Quantitative—cross-sectional	To examine the relationship between exercise type and the stress and depressive symptoms of university students	1. Ball sports (e.g., basketball, floorball, netball, soccer, tennis, volleyball, badminton, ice-hockey and ultimate frisbee) 2. Team	Ball sports vs. aerobic activity vs. weightlifting vs. dancing	1. 451 2. 171 males, 280 females 3. mean age = 22.3 (sd = 2.2)	Stress Perceived Stress Scale (German version) Depressive symptoms The Depression Scale	Among students with high stress (but not low stress), participation in ball sports or dancing was associated with fewer depressive symptoms. Students engaging in weightlifting reported fewer depressive symptoms, but only if they reported having low levels of stress. Aerobic exercise had no moderating effects
1. Hornstrup, 2018 [49] 2. Quantitative-RCT	This study evaluated the effects of regular participation in small-sided team handball training on body composition, osteogenic response, physical performance, and cardiovascular risk factors, as well as well-being and motivation, in young untrained women	1. Handball 2. Team	*Experimental:* 2 × 70 min sessions of team-based handball per week, for 12 weeks *Control:* no exercise control	1. 28 2. All women 3. Mean age: Intervention (*n* = 14): 23.9 (sd = 2.4) Control (*n* = 14): 24.1 (sd = 3.2)	Anxiety Well-being questionnaire (Danish version) Positive well-being Well-being questionnaire (Danish version) Mental energy Well-being questionnaire (Danish version)	There was no effect of the intervention on measures of anxiety or well-being. There was, however, a significant effect of the intervention on mental energy
1. Howie, 2016 [50] 2. Longitudinal (from age 5 to age 20)	The purpose of this study was to identify organized sport trajectories from early childhood to late adolescence. Second the authors explored the associations of these trajectories with physical and mental health outcomes in young adulthood	1. Organized sport 2. Team and/or individual	Sports participation vs no sports participation vs. sports dropouts vs. sports joiners	1. 1679 2. 855/824 3. Age at baseline = 5, age at final assessment = 20	Mental wellbeing Short Form 12-Item Health Survey version 2 Depression Depression Anxiety Stress Scales (DASS-21) Anxiety Depression Anxiety Stress Scales (DASS-21) Stress Depression Anxiety Stress Scales (DASS-21)	Upon examination of the multiple sports participation trajectories, despite differences in physical health outcomes, there were minimal differences in mental health outcomes in adulthood. Of note, males who dropped out of sport in childhood or adolescence had higher depressive symptoms in adulthood
1. Janssen, 2012 [51] 2. Quantitative-experimental (non-randomized)	The present study investigated the influence of physical exercise, cognitive, or Karate training on the cognitive functioning and mental state of older people	1. Karate-Do 2. Group	Each experimental condition involved 20 × 60-min sessions held over 3–6 months *Experimental* (exercise): strength, and flexibility exercises *Experimental* (cognitive): problem solving activities *Experimental* (Karate-Do): Karate-Do training *Control:* Received no training	1. 45 2. 15/30 3. Mean age = 78.8 (sd = 7.0)	Depressive symptoms Centre of Epidemiological Studies Depressions Scale Emotional mental state Center of Epidemiological Studies Depressions Scale	There was an improvement in emotional well-being only in the Karate group This study also showed that the depression score of the control group increased whereas the scores remained the same in the cognitive and exercise groups and decreased in the Karate group
1. Janssen, 2017 [52] 2. Quantitative-RCT (3 groups)	This study investigated the effects of karate versus a mindfulness-based stress reduction (MBSR) intervention on well-being and cognitive functioning in older adults	1. Karate-Do 2. Group	The experimental groups engaged in 2 × 60-min sessions per week for 8 weeks *Experimental* (MBSR): included sitting and walking meditation *Experimental* (Karate-Do): karate-Do training *Control:* received no training	1. 55 2. 21/33 3. Mean age = 63.5 (sd = 5.7) *n* = 25 in karate group, MBSR = 19, control = 22	Subjective well-being Multidimensional Mood Questionnaire (MDBF) Anxiety Hospital Anxiety and Depression Scale-Anxiety (HADS-A) Depression Hospital Anxiety and Depression Scale-Anxiety (HADS-A) Optimism and pessimism The Life Orientation Test–Revised (LOT-R) Subjective mental health The 12-Item Short-Form Health Survey (SF-12-mental) Perceived stress Trier Inventory for Chronic Stress	Participants in the karate condition had significantly greater improvements in subjective mental health and anxiety in comparison to the control and MBSR groups. There were no significant group by time effects for subjective well-being, depression, optimism, pessimism, or perceived stress
1. Jewett, 2014 [53] 2. Quantitative-longitudinal (5 years)	This longitudinal study examined the association between participation in school sport during adolescence and mental health in early adulthood	1. School sport (e.g., basketball, soccer, softball, etc.) 2. Team and individual	Number of years in school sport	1. 853 2. 462 females 3. mean age = 20.39 (sd = .75)	Depressive symptoms Major Depression Inventory (MDI) Perceived stress Single item, assessed on 1–5 scale Mental health Single item, assessed on 1–5 scale	Participation in school sport during adolescence was significantly associated with lower perceived stress, depressive symptoms, and high self-reported mental health in young adulthood
1. Kitchen, 2016 [54] 2.Cross-sectional	The principal objective of this paper is to assess whether participation in ice hockey is associated with an additive health benefit	1. Ice Hockey 2. Team	Stratified by: 'did not play', 'played less than once per week', 'played at least once per week' (regular) vs. 'did not play in the last 3 months'	1. 8250 (for mental health) 8230 (for perceived life stress) 2. – 3. Aged 35 and over	Perceived life stress Single item taken from 'The Community Health Survey (2011/2012) Mental health Single item taken from 'The Community Health Survey (2011/2012)	The results indicate there is no association between playing ice hockey and perceived life stress, nor between ice hockey and self-rated mental health
1. Kim, 2019 [55] 2 Quantitative-ecological momentary assessment (9 weeks)	To better understand the relations between sport consumption (i.e., sport participation, sport spectating, and sport media viewing) and long- and short-term subjective well-being, a study was conducted using ecological momentary assessment and multilevel structural equation modelling	1. Sport participation 2. Not specified	Examination of the short and long-term effects of sports participation	1. 242 2. 49.6% male 3. Mean age = 20.7 (no SE or SD reported) ranging from 18 to 37	Positive affect Positive Affect and Negative Affect Schedule (PANAS) Negative affect Positive Affect and Negative Affect Schedule (PANAS) Life satisfaction Satisfaction with Life Scale	Engagement in sport participation led to perceptions of short-term improvement of positive affect and life satisfaction. There was also evidence that regular sport participation contributes to long-term improvements in positive affect and life satisfaction. There was no significant interaction between sports participation and negative affect Importantly, there was evidence that the short-term effects of sports participation may contribute to long-term improvements in positive affect and life satisfaction
1. Koolhaas, 2018 [56] 2. Cross-sectional	This study assessed the association of total physical activity, walking, cycling, domestic work, sports, and gardening with HRQL in middle-aged and elderly adults	1. Sport (mode not specified) 2. Not specified	Associations between level of participation in sport (low, moderate, high) and MH outcome. Associations calculated separately for 65 and under, and over 65	1. 5554 2. 2356/3198 3. Mean age = 69	Health-related quality of life (mood subscale) Dutch version of the EuroQoL (home interview)	Sports participation was associated with better mood in middle-aged adults and was the physical activity with the most associations domains of (HRQoL) in this age group
1. Marlier, 2015 [36] 2. Quantitative-cross-sectional	The present study aims to uncover how sport participation, physical activity, social capital, and mental health are interrelated by examining these outcomes in one model	1. Sport participation-organized and non-organized 2. Team and individual	Examined how sport participation, total physical activity, social capital, and mental health are interrelated	1. 414 2. 189/225 3. Age range: 18 to 56	Mental health (well-being, more specifically) Goldberg’s General Health Questionnaire (GHQ-12) (self-report)	There was a direct association between sports participation, but not total physical activity, and mental well-being
1. McGraw, 2018 [37] 2. Qualitative-telephone interviews	This qualitative study examined how NFL players and their family members characterized the impact of an NFL career on the mental and emotional health of NFL players	1. NFL (American football) 2. Team	Includes both current and former NFL players, and family members of these players	1. 25 players (23 former and 2 current), 27 family members 2. All male 3. Age not mentioned	Mental health (inclusive of depression, anxiety, loneliness /isolation, and stress) Thematic analysis (interview)	Most of the players and their families reported that the NFL provided emotional benefits as well as improvements to players’ self-esteem. Almost all the players experienced one or more mental health challenge during their career (e.g., depression, anxiety, difficulty controlling temper)
1. Patterson, 2017 [57] 2. Quantitative-RCT	The purpose of the study was to examine the health effects of 8 weeks of recreational badminton in untrained women	1. Badminton 2. Team and individual	*Experimental (Badminton):* 3 1-h sessions per week for 8 weeks. One session per week dedicated to learning skills, with the other 2 dedicated to matches *Experimental (Running):* 3 × 1-h sessions per week for 8 weeks. Sessions involved running around a university at approximately 75% max heart rate *Control:* No exercise control	1. 33 2. All female 3. Mean age = 34.3 (sd = 6.9 years) (range 19–45 years)	Physical self-esteem Physical self-perception profile (PSPP)	Group by time analyses show a significant improvement in the badminton and running groups’ perception of their physical condition. There were no significant group by time interaction effects for sport competence, body attractiveness, strength competence and physical self-worth
1. Purcell, 2020 [39] 2. Quantitative cross-sectional	The purpose of this study was to assess the prevalence and correlates of mental health symptoms in a representative, national sample of elite athletes and to compare rates against published community norms	1. Elite athletes (no singular sport) 2. Not specified	Community rates of psychological variables; 'published community norms'	1. 749 2. 344/405 3.mean age = 24.6 (sd = 6.9)	Mental health symptoms 28-item General Health Questionnaire (GHQ-28) Psychological distress Kessler 10 (K-10) Self-esteem Rosenberg Self-Esteem Scale Maladaptive response to psychological distress (anger and aggression) 2 subscales from the Male Depression Risk Scale (MDRS) Body weight and shape Dissatisfaction weight and shape subscales from the Eating Disorders Examination Questionnaire Satisfaction with life Satisfaction with Life Scale	In the month prior to the survey, approximately 1 in 3 athletes reported mental health symptoms at a level typically requiring treatment by a health professional—a rate significantly higher than community norms. Athletes also reported significantly higher psychological distress than community norms. However, athletes reported greater life satisfaction, self-esteem, and body satisfaction than community norms
1. Sabiston, 2016 [58] 2.Quantitative-longitudinal (5 years)	The purpose of this study was to examine the longitudinal and unique association between number of years of team sport and individual sport participation during adolescence and depressive symptoms during early adulthood	1. Basketball, soccer, football, swimming, baseball, volleyball, hockey, ballet/dance, aerobics classes, ski lessons, and judo/karate 2. Individual and team	Number of years participating in sport; participation in team vs. individual sport	1. 860 2. 54% female 3. Mean age = 20.4 (sd = 0.7) at endpoint	Depressive symptoms Major Depression Inventory (MDI)	The findings of this study indicate adolescents who consistently participated in team sports during secondary school had lower depression scores in early adulthood. Conversely, the number of years of individual sport participation was not related to depressive symptoms in early adulthood
1. Sorenson, 2014 [59] 2. Quantitative-cross-sectional	To assess health-related quality-of-life (HRQL) among current and former National Collegiate Athletic Association student–athletes (SAs)	1. Baseball, basketball, cross-country, American football, golf, rowing, soccer, swimming and diving, tennis, track and field, volleyball, water polo 2. Individual and team	Student athletes (SA) vs. nonathletes (NA)	1. 496 2. 280/215 3. Range = 17–84	Mental health SF-12 mental component score	Current SAs had significantly better mental health than non-athletes. The alumni sample reported better mental health than current students, with age differences significantly greater for NAs
1. Stenner, 2019 [60] 2. Quantitative-cross-sectional	To investigate associations between markers of health and playing golf in an Australian population	1. Golf 2. Not specified	Golfers vs. non-golfers	1. *n* = 9307 2. – 3. Mean age = 48.7 (sd = 17.6)	Health-related quality of life (HRQoL) Question one of the 12-Item Short Form Health Survey (SF-12)	Golfers were more physically active and had 83% higher odds of reporting high HRQoL compared to non-golfers. HRQoL was no longer significantly different between the groups after controlling for physical activity
1. Thorpe, 2014 [40] 2. Qualitative-semi-structured interviews	The aim of the present study was to understand the impact of an Aboriginal community sporting team and its environment on the social, emotional, and physical wellbeing of young Aboriginal men, and to identify barriers and motivators for participation	1. Australian Rules Football 2. Team	–	1. 14 2. All men 3. –	Mental well-being Interviews and group discussion	The players noted that participation gave them a sense of purpose, enjoyment, stress relief, and improvements in self-esteem. They further noted that racism, community conflicts, peer-pressure, and commitment were challenges of playing in the team
1. Tsuji, 2020 [61] 2. Quantitative-cross-sectional	The aim of this study was to identify the prevalence of specific types of sports and exercise groups and the association with self-rated health, depressive symptoms, and frequency of laughter among community-dwelling older people	1. Walking, running, and jogging, fitness exercises, weight exercises, hiking, golf, gateball, dance, yoga, aerobics, petanque, Tai Chi, swimming, aquatics, table tennis, bowling, cycling, tennis, and other 2. Individual and team	Odds ratios of associations between each specific activity and indices of health	1. 131,962 2. 63,465/68497 3. Aged 65 and older	Self-rated health 1-item “How do you feel about your current health status: very good, good, somewhat poor, or poor?” Depressive symptoms 15-item Geriatric Depression Scale (GDS)-GDS Frequency of laughter 1-item “how often do you laugh aloud in your daily life: almost every day, 1 to 5 times a week, one to 3 × per month, or almost none?”	Playing golf in a group was related to better self-rated health, less experience of depressive symptoms, and a higher frequency of laughter compared to not playing golf in a group Walking in a group was related to better self-rated health for females, and to less experience of depressive symptoms and a higher frequency of laughter in males and females Walking in a group was also related to a greater likelihood of reporting excellent self-rated health for females, and to more laughter and lower depressive symptoms in both males and females
1. Yamakita, 2015 [62] 2. Quantitative-cross-sectional	The purpose of this study was to identify the demographic and biological, psychosocial, behavioral, social, and cultural, and environmental correlates of sports group participation among Japanese older adults	1. Sports group or club 2. Not specified	Regular participation vs. non-regular participation	1. 78,002 2. 37,772/40230 3. Mean age = 73.5 (sd = 6.1)	Depression Short version of the Geriatric Depression Scale–15	Irregular or no participation in sports was associated with mild to severe depression

### Effects of sports participation on psychological well-being, ill-being, and social outcomes

#### Cross-sectional evidence

This review included 14 studies reporting on the cross-sectional relationship between sports participation and psychological and/or social outcomes. Sample sizes range from *n* = 414 to *n* = 131,962 with a total of *n* = 239,394 adults included across the cross-sectional studies.

The cross-sectional evidence generally supports that participation in sport, and especially team sports, is associated with greater mental health and psychological wellbeing in adults compared to non-participants [36, 59]; and that higher frequency of sports participation and/or sport played at a higher level of competition, are also linked to lower levels of mental distress in adults . This was not the case for one specific study involving ice hockey players aged 35 and over, with Kitchen and Chowhan (2016) Kitchen and Chowhan (2016) reporting no relationship between participation in ice hockey and either mental health, or perceived life stress [54]. There is also some evidence to support that previous participation in sports (e.g., during childhood or young adulthood) is linked to better mental health outcomes later in life, including improved mental well-being and lower mental distress [59], even after controlling for age and current physical activity.

Compared to published community data for adults, elite or high-performance adult athletes demonstrated higher levels of body satisfaction, self-esteem, and overall life satisfaction [39]; and reported reduced tendency to respond to distress with anger and depression. However, rates of psychological distress were higher in the elite sport cohort (compared to community norms), with nearly 1 in 5 athletes reporting ‘high to very high’ distress, and 1 in 3 reporting poor mental health symptoms at a level warranting treatment by a health professional in one study (*n* = 749) [39].

Four studies focused on the associations between physical activity and sports participation and mental health outcomes in older adults. Physical activity was associated with greater quality of life [56], with the relationship strongest for those participating in sport in middle age, and for those who cycled in later life (> 65) [56]. Group physical activities (e.g., walking groups) and sports (e.g., golf) were also significantly related to excellent self-rated health, low depressive symptoms, high health-related quality of life (HRQoL) and a high frequency of laughter in males and females [60, 61]. No participation or irregular participation in sport was associated with symptoms of mild to severe depression in older adults [62].

Several cross-sectional studies examined whether the effects of physical activity varied by type (e.g., total physical activity vs. sports participation). In an analysis of 1446 young adults (mean age = 18), total physical activity, moderate-to-vigorous physical activity, and team sport were independently associated with mental health [46]. Relative to individual physical activity, after adjusting for covariates and moderate-to-vigorous physical activity (MVPA), only team sport was significantly associated with improved mental health. Similarly, in a cross-sectional analysis of Australian women, Eime, Harvey, Payne (2014) reported that women who engaged in club and team-based sports (tennis or netball) reported better mental health and life satisfaction than those who engaged in individual types of physical activity [47]. Interestingly, there was no relationship between the amount of physical activity and either of these outcomes, suggesting that other qualities of sports participation contribute to its relationship to mental health and life satisfaction. There was also some evidence to support a relationship between exercise type (ball sports, aerobic activity, weightlifting, and dancing), and mental health amongst young adults (mean age 22 years) [48], with ball sports and dancing related to fewer symptoms of depression in students with high stress; and weightlifting related to fewer depressive symptoms in weightlifters exhibiting low stress.

#### Longitudinal evidence

Eight studies examined the longitudinal relationship between sports participation and either mental health and/or social outcomes. Sample sizes range from *n* = 113 to *n* = 1679 with a total of *n* = 7022 adults included across the longitudinal studies.

Five of the included longitudinal studies focused on the relationship between sports participation in childhood or adolescence and mental health in young adulthood. There is evidence that participation in sport in high-school is protective of future symptoms of anxiety (including panic disorder, generalised anxiety disorder, social phobia, and agoraphobia) [42]. Specifically, after controlling for covariates (including current physical activity), the number of years of sports participation in high school was shown to be protective of symptoms of panic and agoraphobia in young adulthood, but not protective of symptoms of social phobia or generalized anxiety disorder [42]. A comparison of individual or team sports participation also revealed that participation in either context was protective of panic disorder symptoms, while only team sport was protective of agoraphobia symptoms, and only individual sport was protective of social phobia symptoms. Furthermore, current and past sports team participation was shown to negatively relate to adult depressive symptoms [43]; drop out of sport was linked to higher depressive symptoms in adulthood compared to those with maintained participation [9, 22, 63]; and consistent participation in team sports (but not individual sport) in adolescence was linked to higher self-rated mental health, lower perceived stress and depressive symptoms, and lower depression scores in early adulthood [53, 58].

Two longitudinal studies [35, 55], also investigated the association between team and individual playing context and mental health. Dore and colleagues [35] reported that compared to individual activities, being active in informal groups (e.g., yoga, running groups) or team sports was associated with better mental health, fewer depressive symptoms and higher social connectedness – and that involvement in team sports was related to better mental health regardless of physical activity volume. Kim and James [55] discovered that sports participation led to both short and long-term improvements in positive affect and life satisfaction.

A study on social outcomes related to mixed martial-arts (MMA) and Brazilian jiu-jitsu (BJJ) showed that both sports improved practitioners’ self-control and pro-social behavior, with greater improvements seen in the BJJ group [62]. Notably, while BJJ reduced participants’ reported aggression, there was a slight increase in MMA practitioners, though it is worth mentioning that individuals who sought out MMA had higher levels of baseline aggression.

#### Experimental evidence

Six of the included studies were experimental or quasi-experimental. Sample sizes ranged from *n* = 28 to *n* = 55 with a total of *n* = 239 adults included across six longitudinal studies. Three studies involved a form of martial arts (such as judo and karate) [45, 51, 52], one involved a variety of team sports (such as netball, soccer, and cricket) [34], and the remaining two focused on badminton [57] and handball [49].

Brinkley and colleagues [34] reported significant effects on interpersonal communication (but not vitality, social cohesion, quality of life, stress, or interpersonal relationships) for participants (*n* = 40) engaging in a 12-week workplace team sports intervention. Also using a 12-week intervention, Hornstrup et al. [49] reported a significant improvement in mental energy (but not well-being or anxiety) in young women (mean age = 24; *n* = 28) playing in a handball program. Patterns et al. [57] showed that in comparison to no exercise, participation in an 8-week badminton or running program had no significant improvement on self-esteem, despite improvements in perceived and actual fitness levels.

Three studies examined the effect of martial arts on the mental health of older adults (mean ages 79 [52], 64 [51], and 70 [45] years). Participation in Karate-Do had positive effects on overall mental health, emotional wellbeing, depression and anxiety when compared to other activities (physical, cognitive, mindfulness) and a control group [51, 52]. Ciaccioni et al. [45] found that a Judo program did not affect either the participants’ mental health or their body satisfaction, citing a small sample size, and the limited length of the intervention as possible contributors to the findings.

#### Qualitative evidence

Three studies interviewed current or former sports players regarding their experiences with sport. Chinkov and Holt [41] reported that jiu-jitsu practitioners (mean age 35 years) were more self-confident in their lives outside of the gym, including improved self-confidence in their interactions with others because of their training. McGraw and colleagues [37] interviewed former and current National Football League (NFL) players and their families about its impact on the emotional and mental health of the players. Most of the players reported that their NFL career provided them with social and emotional benefits, as well as improvements to their self-esteem even after retiring. Though, despite these benefits, almost all the players experienced at least one mental health challenge during their career, including depression, anxiety, or difficulty controlling their temper. Some of the players and their families reported that they felt socially isolated from people outside of the national football league.

Through a series of semi-structured interviews and focus groups, Thorpe, Anders [40] investigated the impact of an Aboriginal male community sporting team on the health of its players. The players reported they felt a sense of belonging when playing in the team, further noting that the social and community aspects were as important as the physical health benefits. Participating in the club strengthened the cultural identity of the players, enhancing their well-being. The players further noted that participation provided them with enjoyment, stress relief, a sense of purpose, peer support, and improved self-esteem. Though they also noted challenges, including the presence of racism, community conflict, and peer-pressure.

### Quality of studies

Full details of our risk of bias (ROB) results are provided in Supplementary Material A. Of the three qualitative studies assessed using the Critical Appraisal Skills Program (CASP), all three were deemed to have utilised and reported appropriate methodological standards on at least 8 of the 10 criteria. Twenty studies were assessed using the Quality Assessment Tool for Observational Cohort and Cross-Sectional Studies, with all studies clearly reporting the research question/s or objective/s and study population. However, only four studies provided a justification for sample size, and less than half of the studies met quality criteria for items 6, 7, 9, or 10 (and items 12 and 13 were largely not applicable). Of concern, only four of the observational or cohort studies were deemed to have used clearly defined, valid, and reliable exposure measures (independent variables) and implemented them consistently across all study participants. Six studies were assessed using the Quality Assessment of Controlled Intervention Studies, with three studies described as a randomized trial (but none of the three reported a suitable method of randomization, concealment of treatment allocation, or blinding to treatment group assignment). Three studies showed evidence that study groups were similar at baseline for important characteristics and an overall drop-out rate from the study < 20%. Four studies reported high adherence to intervention protocols (with two not reporting) and five demonstrated that.study outcomes were assessed using valid and reliable measures and implemented consistently across all study participants. Importantly, researchers did not report or have access to validated instruments for assessing sport participation or physical activity amongst adults, though most studies provided psychometrics for their mental health outcome measure/s. Only one study reported that the sample size was sufficiently powered to detect a difference in the main outcome between groups (with ≥ 80% power) and that all participants were included in the analysis of results (intention-to-treat analysis). In general, the methodological quality of the six randomised studies was deemed low.

## Discussion

Initially, our discussion will focus on the review findings regarding sports participation and well-being, ill-being, and psychological health. However, the heterogeneity and methodological quality of the included research (especially controlled trials) should be considered during the interpretation of our results. Considering our findings, the Mental Health through Sport conceptual model for adults will then be presented and discussed and study limitations outlined.

### Sports participation and psychological well-being

In summary, the evidence presented here indicates that for adults, sports participation is associated with better overall mental health [36, 46, 47, 59], mood [56], higher life satisfaction [39, 47], self-esteem [39], body satisfaction [39], HRQoL [60], self-rated health [61], and frequency of laughter [61]. Sports participation has also shown to be predictive of better psychological wellbeing over time [35, 53], higher positive affect [55], and greater life satisfaction [55]. Furthermore, higher frequency of sports participation and/or sport played at a higher level of competition, have been linked to lower levels of mental distress, higher levels of body satisfaction, self-esteem, and overall life satisfaction in adults [39].

Despite considerable heterogeneity of sports type, cross-sectional and experimental research indicate that team-based sports participation, compared to individual sports and informal group physical activity, has a more positive effect on mental energy [49], physical self-perception [57], and overall psychological health and well-being in adults, regardless of physical activity volume [35, 46, 47]. And, karate-do benefits the subjective well-being of elderly practitioners [51, 52]. Qualitative research in this area has queried participants’ experiences of jiu-jitsu, Australian football, and former and current American footballers. Participants in these sports reported that their participation was beneficial for psychological well-being [37, 40, 41], improved self-esteem [37, 40, 41], and enjoyment [37].

### Sports participation and psychological ill-being

Of the included studies, *n* = 19 examined the relationship between participating in sport and psychological ill-being. In summary, there is consistent evidence that sports participation is related to lower depression scores [43, 48, 61, 62]. There were mixed findings regarding psychological stress, where participation in childhood (retrospectively assessed) was related to lower stress in young adulthood [41], but no relationship was identified between recreational hockey in adulthood and stress [54]. Concerning the potential impact of competing at an elite level, there is evidence of higher stress in elite athletes compared to community norms [39]. Further, there is qualitative evidence that many current or former national football league players experienced at least one mental health challenge, including depression, anxiety, difficulty controlling their temper, during their career [37].

Evidence from longitudinal research provided consistent evidence that participating in sport in adolescence is protective of symptoms of depression in young adulthood [43, 53, 58, 63], and further evidence that participating in young adulthood is related to lower depressive symptoms over time (6 months) [35]. Participation in adolescence was also protective of manifestations of anxiety (panic disorder and agoraphobia) and stress in young adulthood [42], though participation in young adulthood was not related to a more general measure of anxiety [35] nor to changes in negative affect [55]). The findings from experimental research were mixed. Two studies examined the effect of karate-do on markers of psychological ill-being, demonstrating its capacity to reduce anxiety [52], with some evidence of its effectiveness on depression [51]. The other studies examined small-sided team-based games but showed no effect on stress or anxiety [34, 49]. Most studies did not differentiate between team and individual sports, though one study found that adolescents who participated in team sports (not individual sports) in secondary school has lower depression scores in young adulthood [58].

### Sports participation and social outcomes

Seven of the included studies examined the relationship between sports participation and social outcomes. However, very few studies examined social outcomes or tested a social outcome as a potential mediator of the relationship between sport and mental health. It should also be noted that this body of evidence comes from a wide range of sport types, including martial arts, professional football, and workplace team-sport, as well as different methodologies. Taken as a whole, the evidence shows that participating in sport is beneficial for several social outcomes, including self-control [50], pro-social behavior [50], interpersonal communication [34], and fostering a sense of belonging [40]. Further, there is evidence that group activity, for example team sport or informal group activity, is related to higher social connectedness over time, though analyses showed that social connectedness was not a mediator for mental health [35].

There were conflicting findings regarding social effects at the elite level, with current and former NFL players reporting that they felt socially isolated during their career [37], whilst another study reported no relationship between participation at the elite level and social dysfunction [39]. Conversely, interviews with a group of indigenous men revealed that they felt as though participating in an all-indigenous Australian football team provided them with a sense of purpose, and they felt as though the social aspect of the game was as important as the physical benefits it provides [40].

### Mental health through sport conceptual model for adults

**Fig. 2:**
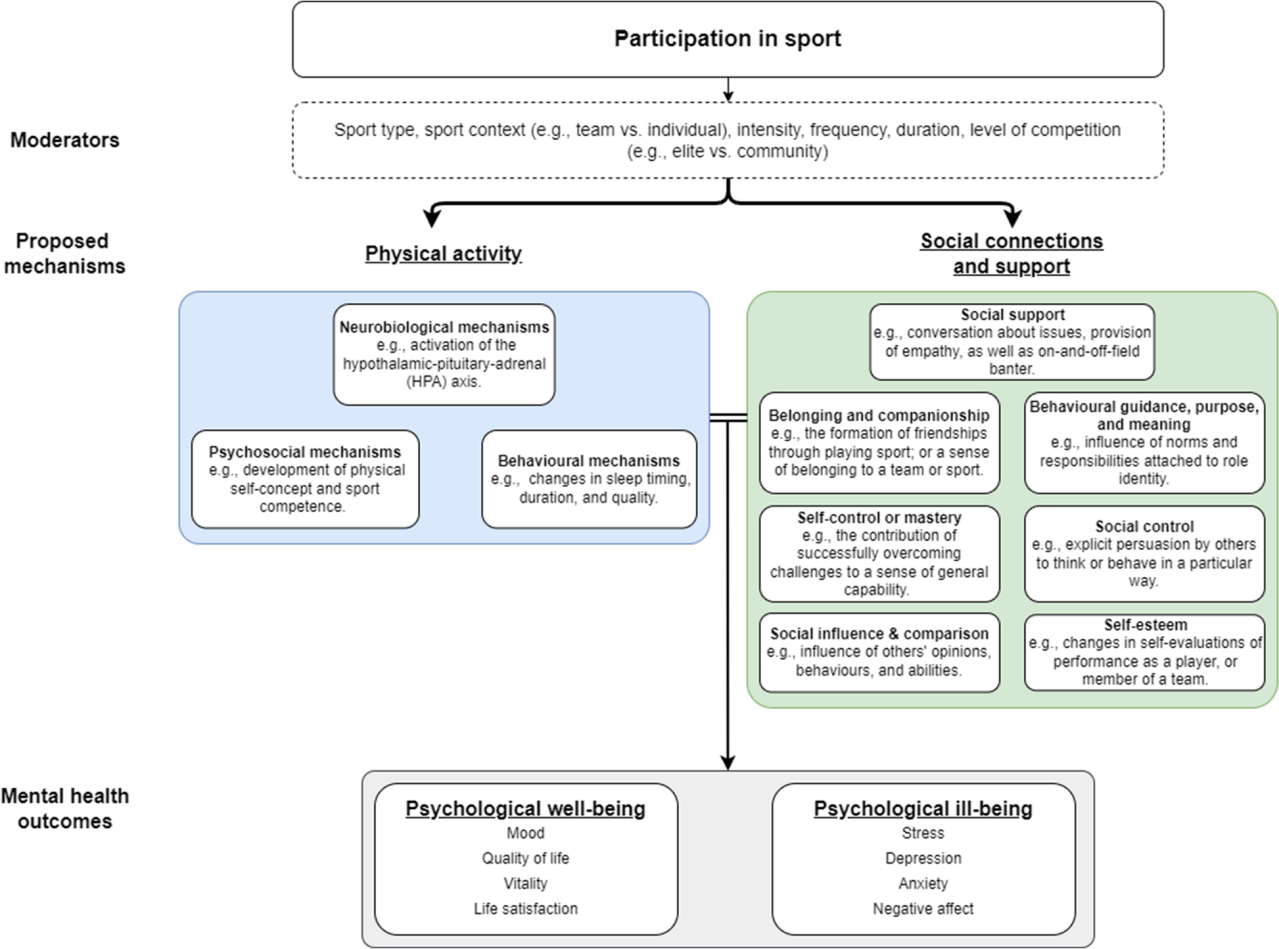
The Mental Health through Sport conceptual model

The ‘Health through Sport’ model provides a depiction of the determinants and benefits of sports participation [31]. The model recognises that the physical, mental, and social benefits of sports participation vary by the context of sport (e.g., individual vs. team, organized vs. informal). To identify the elements of sport which contribute to its effect on mental health outcomes, we describe the ‘Mental Health through Sport’ model (Fig. 2). The model proposes that the social and physical elements of sport each provide independent, and likely synergistic contributions to its overall influence on mental health.

The model describes two key pathways through which sport may influence mental health: physical activity, and social relationships and support. Several likely moderators of this effect are also provided, including sport type, intensity, frequency, context (team vs. individual), environment (e.g., indoor vs. outdoor), as well as the level of competition (e.g., elite vs. amateur).

The means by which the physical activity component of sport may influence mental health stems from the work of Lubans et al., who propose three key groups of mechanisms: neurobiological, psychosocial, and behavioral [64]. Processes whereby physical activity may enhance psychological outcomes via changes in the structural and functional composition of the brain are referred to as neurobiological mechanisms [65, 66]. Processes whereby physical activity provides opportunities for the development of self-efficacy, opportunity for mastery, changes in self-perceptions, the development of independence, and for interaction with the environment are considered psychosocial mechanisms. Lastly, processes by which physical activity may influence behaviors which ultimately affect psychological health, including changes in sleep duration, self-regulation, and coping skills, are described as behavioral mechanisms.

Playing sport offers the opportunity to form relationships and to develop a social support network, both of which are likely to influence mental health. Thoits [29] describes 7 key mechanisms by which social relationships and support may influence mental health: social influence/social comparison; social control; role-based purpose and meaning (mattering); self-esteem; sense of control; belonging and companionship; and perceived support availability [29]. These mechanisms and their presence within a sporting context are elaborated below.

Subjective to the attitudes and behaviors of individuals in a group, *social influence and comparison* may facilitate protective or harmful effects on mental health. Participants in individual or team sport will be influenced and perhaps steered by the behaviors, expectations, and norms of other players and teams. When individual’s compare their capabilities, attitudes, and values to those of other participants, their own behaviors and subsequent health outcomes may be affected. When others attempt to encourage or discourage an individual to adopt or reject certain health practices, *social control* is displayed [29]. This may evolve as strategies between players (or between players and coach) are discussion and implemented. Likewise, teammates may try to motivate each another during a match to work harder, or to engage in specific events or routines off-field (fitness programs, after game celebrations, attending club events) which may impact current and future physical and mental health.

Sport may also provide *behavioral* *guidance, purpose, and meaning* to its participants. Role identities (positions within a social structure that come with reciprocal obligations), often formed as a consequence of social ties formed through sport. Particularly in team sports, participants come to understand they form an integral part of the larger whole, and consequently, they hold certain responsibility in ensuring the team’s success. They have a commitment to the team to, train and play, communicate with the team and a potential responsibility to maintain a high level of health, perform to their capacity, and support other players. As a source of behavioral guidance and of purpose and meaning in life, these identities are likely to influence mental health outcomes amongst sport participants.

An individual’s level of *self-esteem* may be affected by the social relationships and social support provided through sport; with improved perceptions of capability (or value within a team) in the sporting domain likely to have positive impact on global self-esteem and sense of worth [64]. The unique opportunities provided through participation in sport, also allow individuals to develop new skills, overcome challenges, and develop their sense of *self-control or mastery*. Working towards and finding creative solutions to challenges in sport facilitates a sense of mastery in participants. This sense of mastery may translate to other areas of life, with individual’s developing the confidence to cope with varied life challenges. For example, developing a sense of mastery regarding capacity to formulate new / creative solutions when taking on an opponent in sport may result in greater confidence to be creative at work. Social relationships and social support provided through sport may also provide participants with a source of *belonging and companionship.* The development of connections (on and off the field) to others who share common interests, can build a sense of belonging that may mediate improvements in mental health outcomes. *Social support* is often provided emotionally during expressions of trust and care; instrumentally via tangible assistance; through information such as advice and suggestions; or as appraisal such feedback. All forms of social support provided on and off the field contribute to a more generalised sense of perceived support that may mediate the effect of social interaction on mental health outcomes.

Participation in sport may influence mental health via some combination of the social mechanisms identified by Thoits, and the neurobiological, psychosocial, and behavioral mechanisms stemming from physical activity identified by Lubans [29, 64]. The exact mechanisms through which sport may confer psychological benefit is likely to vary between sports, as each sport varies in its physical and social requirements. One must also consider the social effects of sports participation both on and off the field. For instance, membership of a sporting team and/or club may provide a sense of identity and belonging—an effect that persists beyond the immediacy of playing the sport and may have a persistent effect on their psychological health. Furthermore, the potential for team-based activity to provide additional benefit to psychological outcomes may not just be attributable to the differences in social interactions, there are also physiological differences in the requirements for sport both within (team vs. team) and between (team vs. individual) categories that may elicit additional improvements in psychological outcomes. For example, evidence supports that exercise intensity moderates the relationship between physical activity and several psychological outcomes—supporting that sports performed at higher intensity will be more beneficial for psychological health.

### Limitations and recommendations

There are several limitations of this review worthy of consideration. Firstly, amongst the included studies there was considerable heterogeneity in study outcomes and study methodology, and self-selection bias (especially in non-experimental studies) is likely to influence study findings and reduce the likelihood that study participants and results are representative of the overall population. Secondly, the predominately observational evidence included in this and Eime’s prior review enabled us to identify the positive relationship between sports participation and social and psychological health (and examine directionality)—but more experimental and longitudinal research is required to determine causality and explore potential mechanisms responsible for the effect of sports participation on participant outcomes. Additional qualitative work would also help researchers gain a better understanding of the relationship between specific elements of the sporting environment and mental health and social outcomes in adult participants. Thirdly, there were no studies identified in the literature where sports participation involved animals (such as equestrian sports) or guns (such as shooting sports). Such studies may present novel and important variables in the assessment of mental health benefits for participants when compared to non-participants or participants in sports not involving animals/guns—further research is needed in this area. Our proposed conceptual model also identifies several pathways through which sport may lead to improvements in mental health—but excludes some potentially negative influences (such as poor coaching behaviors and injury). And our model is not designed to capture all possible mechanisms, creating the likelihood that other mechanisms exist but are not included in this review. Additionally, an interrelationship exits between physical activity, mental health, and social relationships, whereby changes in one area may facilitate changes in the other/s; but for the purpose of this study, we have focused on how the physical and social elements of sport may mediate improvements in psychological outcomes. Consequently, our conceptual model is not all-encompassing, but designed to inform and guide future research investigating the impact of sport participation on mental health.

## Conclusion

The findings of this review endorse that participation in sport is beneficial for psychological well-being, indicators of psychological ill-being, and social outcomes in adults. Furthermore, participation in team sports is associated with better psychological and social outcomes compared to individual sports or other physical activities. Our findings support and add to previous review findings [1]; and have informed the development of our ‘Mental Health through Sport’ conceptual model for adults which presents the potential mechanisms by which participation in sport may affect mental health.

## Supplementary Information

**Additional file 1: Supplementary Table A.** Risk of bias.

**Additional file 2: Supplementary Table B.** PRISMA Checklist.  

## Data Availability

The datasets used and/or analyzed during the current study are available from the corresponding author on reasonable request.

## References

[1] Eime RM, Young JA, Harvey JT, Charity MJ, Payne WR (2013). A systematic review of the psychological and social benefits of participation in sport for adults: informing development of a conceptual model of health through sport. Int J Behav Nutr Phys Act.

[2] Ishihara T, Nakajima T, Yamatsu K, Okita K, Sagawa M, Morita N. Relationship of participation in specific sports to academic performance in adolescents: a 2-year longitudinal study. Scand J Med Sci Sports. 2020. 10.1111/sms.13703 32350922

[3] Cope E, Bailey R, Pearce G (2013). Why do children take part in, and remain involved in sport?: implications for children’s sport coaches. Int J Coach Sci.

[4] Harrison PA, Narayan G (2003). Differences in behavior, psychological factors, and environmental factors associated with participation in school sports and other activities in adolescence. J Sch Health.

[5] Allender S, Cowburn G, Foster C (2006). Understanding particpation in sport and physical activity among children and adults: a review of qualitative studies. Health Educ Res.

[6] Adachi P, Willoughby T (2014). It's not how much you play, but how much you enjoy the game: The longitudinal associations between adolescents' self-esteem and the frequency versus enjoyment of involvement in sports. J Youth Adolesc.

[7] Malm C, Jakobsson J, Isaksson A (2019). Physical activity and sports-real health benefits: a review with insight into the public health of Sweden. Sports (Basel, Switzerland).

[8] Mills K, Dudley D, Collins NJ (2019). Do the benefits of participation in sport and exercise outweigh the negatives? An academic review. Best Pract Res Clin Rheumatol.

[9] Howie EK, Guagliano JM, Milton K, Vella SA, Gomersall SR,Kolbe-Alexander TL,  (2020). Ten research priorities related to youth sport, physical activity, and health.

[10] Vella SA, Swann C, Allen MS, Schweickle MJ, Magee CA (2017). Bidirectional associations between sport involvement and mental health in adolescence. Med Sci Sports Exerc.

[11] Andersen MH, Ottesen L, Thing LF (2019). The social and psychological health outcomes of team sport participation in adults: An integrative review of research. Scand J Public Health.

[12] Rice SM, Purcell R, De Silva S, Mawren D, McGorry PD, Parker AG (2016). The mental health of elite athletes: a narrative systematic review. Sports medicine (Auckland, NZ).

[13] Hulteen RM, Smith JJ, Morgan PJ, Barnett LM, Hallal PC, Colyvas K (2017). Global participation in sport and leisure-time physical activities: a systematic review and meta-analysis. Prev Med.

[14] Eime RM, Harvey J, Charity M, Westerbeek H (2020). Longitudinal Trends in Sport Participation and Retention of Women and Girls. Front Sports Act Living.

[15] Brooke HL, Corder K, Griffin SJ, van Sluijs EMF (2014). Physical activity maintenance in the transition to adolescence: a longitudinal study of the roles of sport and lifestyle activities in british youth. PLoS ONE.

[16] Coll CVN, Knuth AG, Bastos JP, Hallal PC, Bertoldi AD (2014). Time trends of physical activity among Brazilian adolescents over a 7-year period. J Adolesc Health.

[17] Klostermann C, Nagel S (2012). Changes in German sport participation: Historical trends in individual sports. Int Rev Sociol Sport.

[18] Eime RM, Harvey J, Charity M (2020). Sport participation settings: where and 'how' do Australians play sport?. BMC Public Health.

[19] Lim SY, Warner S, Dixon M, Berg B, Kim C, Newhouse-Bailey M (2011). Sport Participation Across National Contexts: A Multilevel Investigation of Individual and Systemic Influences on Adult Sport Participation. Eur Sport Manag Q.

[20] World Health Organisation (2013). Mental Health Action Plan 2013–2020.

[21] Keyes C (2014). Bridging Occupational, Organizational and Public Health.

[22] Ryff C, Love G, Urry H, Muller D, Rosenkranz M, Friedman E (2006). Psychological well-being and ill-being: Do they have distinct or mirrored biological correlates?. Psychother Psychosom.

[23] Australian Government (2013). Social and emotional wellbeing: Development of a children’s headline indicator information paper.

[24] Global Burden of Disease Injury IP (2018). Collaborators, Global, regional, and national incidence, prevalence, and years lived with disability for 354 diseases and injuries for 195 countries and territories, 1990–2017: a systematic analysis for the Global Burden of Disease Study 2017. Lancet.

[25] World Health Organisation. Mental disorders: Fact sheet 2019 [Available from: https://www.who.int/news-room/fact-sheets/detail/mental-disorders.

[26] Mental Health [Internet]. 2018 [cited 12 March 2021]. Available from: https://ourworldindata.org/mental-health' [Online Resource].

[27] Newman MG, Zainal NH (2020). The value of maintaining social connections for mental health in older people. The Lancet Public Health.

[28] Eime RM, Harvey JT, Brown WJ, Payne WR (2010). Does sports club participation contribute to health-related quality of life?. Med Sci Sports Exerc.

[29] Thoits PA (2011). Mechanisms linking social ties and support to physical and mental health. J Health Soc Behav.

[30] Page MJ, McKenzie JE, Bossuyt PM, Boutron I, Hoffmann TC, Mulrow CD (2021). The PRISMA 2020 statement: an updated guideline for reporting systematic reviews. PLoS Med.

[31] Eime RM, Young JA, Harvey JT, Charity MJ, Payne WR (2013). A systematic review of the psychological and social benefits of participation in sport for adults: informing development of a conceptual model of health through sport. Int J Behav Nutr Phys Act.

[32] Critical Appraisal Skills Programme. CASP Qualitative Studies Checklist2019 1/12/2021]. Available from: https://casp-uk.b-cdn.net/wp-content/uploads/2018/01/CASP-Qualitative-Checklist-2018.pdf.

[33] National Institutes from Health. Quality assessment tool for observational cohort and cross-sectional studies 2014 [Available from: https://www.nhlbi.nih.gov/health-topics/study-quality-assessment-tools.

[34] Brinkley A, McDermott H, Grenfell-Essam R (2017). It's time to start changing the game: a 12-week workplace team sport intervention study. Sports Med Open.

[35] Doré I, O'Loughlin JL, Schnitzer ME, Datta GD, Fournier L (2018). The longitudinal association between the context of physical activity and mental health in early adulthood. Ment Health Phys Act.

[36] Marlier M, Van Dyck D, Cardon G, De Bourdeaudhuij I, Babiak K, Willem A. Interrelation of sport participation, physical activity, social capital and mental health in disadvantaged communities: A sem-analysis. PLoS ONE [Internet]. 2015; 10(10):[e0140196 p.]. Available from: http://ezproxy.newcastle.edu.au/login?url=http://ezproxy.newcastle.edu.au/login?url=http://ovidsp.ovid.com/ovidweb.cgi?T=JS&CSC=Y&NEWS=N&PAGE=fulltext&D=med12&AN=26451731. 10.1371/journal.pone.0140196 PMC4599734 26451731

[37] McGraw SA, Deubert CR, Lynch HF, Nozzolillo A, Taylor L, Cohen I (2018). Life on an emotional roller coaster: NFL players and their family members' perspectives on player mental health. J Clin Sport Psychol.

[38] Mickelsson T. Modern unexplored martial arts – what can mixed martial arts and Brazilian Jiu-Jitsu do for youth development?. Eur J Sport Sci. 2020;20(3):386–93. 10.1080/17461391.2019.1629180. 10.1080/17461391.2019.1629180 31167632

[39] Purcell R, Rice S, Butterworth M, Clements M. Rates and Correlates of Mental Health Symptoms in Currently Competing Elite Athletes from the Australian National High-Performance Sports System. Sports Med. 2020. 10.1007/s40279-020-01266-z 32026315

[40] Thorpe A, Anders W, Rowley K (2014). The community network: an Aboriginal community football club bringing people together. Aust J Prim Health.

[41] Appelqvist-Schmidlechner K, Vaara J, Hakkinen A, Vasankari T, Makinen J, Mantysaari M (2018). Relationships between youth sports participation and mental health in young adulthood among Finnish males. Am J Health Promot.

[42] Ashdown-Franks G, Sabiston CM, Solomon-Krakus S, O'Loughlin JL (2017). Sport participation in high school and anxiety symptoms in young adulthood. Ment Health Phys Act.

[43] Brunet J, Sabiston CM, Chaiton M, Barnett TA, O'Loughlin E, Low NC (2013). The association between past and current physical activity and depressive symptoms in young adults: a 10-year prospective study. Ann Epidemiol.

[44] Chinkov AE, Holt NL. Implicit transfer of life skills through participation in Brazilian Jiu-jitsu. J Appl Sport Psychol. 2016;28(2):139–53. 10.1080/10413200.2015.1086447.

[45] Ciaccioni S, Capranica L, Forte R, Chaabene H, Pesce C, Condello G (2019). Effects of a judo training on functional fitness, anthropometric, and psychological variables in old novice practitioners. J Aging Phys Act.

[46] Doré I, O'Loughlin JL, Beauchamp G, Martineau M, Fournier L (2016). Volume and social context of physical activity in association with mental health, anxiety and depression among youth. Prev Med.

[47] Eime R, Harvey J, Payne W (2014). Dose-response of women's health-related quality of life (HRQoL) and life satisfaction to physical activity. J Phys Act Health.

[48] Gerber M, Brand S, Elliot C, Holsboer-Trachsler E, Pühse U (2014). Aerobic exercise, ball sports, dancing, and weight Lifting as moderators of the relationship between Stress and depressive symptoms: an exploratory cross-sectional study with Swiss university students. Percept Mot Skills.

[49] Hornstrup T, Wikman JM, Fristrup B, Póvoas S, Helge EW, Nielsen SH (2018). Fitness and health benefits of team handball training for young untrained women—a cross-disciplinary RCT on physiological adaptations and motivational aspects. J Sport Health Sci.

[50] Mickelsson T. Modern unexplored martial arts–what can mixed martial arts and Brazilian Jiu-Jiutsu do for youth development? Eur J Sport Sci. 2019. 10.1080/17461391.2019.1629180 31167632

[51] Jansen P, Dahmen-Zimmer K (2012). Effects of cognitive, motor, and karate training on cognitive functioning and emotional well-being of elderly people. Front Psychol.

[52] Jansen P, Dahmen-Zimmer K, Kudielka BM, Schulz A (2017). Effects of karate training versus mindfulness training on emotional well-being and cognitive performance in later life. Res Aging.

[53] Jewett R, Sabiston CM, Brunet J, O'Loughlin EK, Scarapicchia T, O'Loughlin J (2014). School sport participation during adolescence and mental health in early adulthood. J Adolesc Health.

[54] Kitchen P, Chowhan J (2016). Forecheck, backcheck, health check: the benefits of playing recreational ice hockey for adults in Canada. J Sports Sci.

[55] Kim J, James JD. Sport and happiness: Understanding the relations among sport consumption activities, long-and short-term subjective well-being, and psychological need fulfillment. J Sport Manage. 2019.

[56] Koolhaas CH, Dhana K, Van Rooij FJA, Schoufour JD, Hofman A, Franco OH (2018). Physical activity types and health-related quality of life among middle-aged and elderly adults: the Rotterdam study. J Nutr Health Aging.

[57] Patterson S, Pattison J, Legg H, Gibson AM, Brown N (2017). The impact of badminton on health markers in untrained females. J Sports Sci.

[58] Sabiston CM, Jewett R, Ashdown-Franks G, Belanger M, Brunet J, O’Loughlin E (2016). Number of years of team and individual sport participation during adolescence and depressive symptoms in early adulthood. J Sport Exerc Psychol.

[59] Sorenson SC, Romano R, Scholefield RM, Martin BE, Gordon JE, Azen SP (2014). Holistic life-span health outcomes among elite intercollegiate student-athletes. J Athl Train.

[60] Stenner B, Mosewich AD, Buckley JD, Buckley ES. Associations between markers of health and playing golf in an Australian population. BMJ Open Sport Exerc Med. 2019;5(1). 10.1136/bmjsem-2019-000517 PMC6539163 31191971

[61] Tsuji T, Kanamori S, Saito M, Watanabe R, Miyaguni Y, Kondo K (2020). Specific types of sports and exercise group participation and socio-psychological health in older people. J Sports Sci.

[62] Yamakita M, Kanamori S, Kondo N, Kondo K (2015). Correlates of regular participation in sports groups among Japanese older adults: JAGES cross–sectional study. PLoS ONE.

[63] Howie EK, McVeigh JA, Smith AJ, Straker LM (2016). Organized sport trajectories from childhood to adolescence and health associations. Med Sci Sports Exerc.

[64] Chinkov AE, Holt NL (2016). Implicit transfer of life skills through participation in Brazilian Jiu-Jitsu. J Appl Sport Psychol.

[65] Lubans D, Richards J, Hillman C, Faulkner G, Beauchamp M, Nilsson M (2016). Physical activity for cognitive and mental health in youth: a systematic review of mechanisms. Pediatrics.

[66] Lin TW, Kuo YM (2013). Exercise benefits brain function: the monoamine connection. Brain Sci.

[67] Dishman RK, O'Connor PJ (2009). Lessons in exercise neurobiology: the case of endorphins. Ment Health Phys Act.

